# Nasolabial Fold Hyaluronic Acid Injection‐Induced Granulomatous Perioral Dermatitis‐Like Lesions: A Case Report

**DOI:** 10.1111/jocd.70089

**Published:** 2025-02-28

**Authors:** Xiaoyu Sun, Wenjia Yang

**Affiliations:** ^1^ Department of Dermatology, Hangzhou Third People's Hospital Affiliated to Zhejiang Chinese Medical University Hangzhou P.R. China

To the Editor,

Hyaluronic acid (HA), a hydrophilic biopolymer, is widely used in medical aesthetics as a non‐surgical filler due to its role in tissue regeneration, anti‐inflammation, and immune modulation [1]. Although generally safe, the increasing popularity of HA injections has revealed certain adverse effects, including vascular occlusion and delayed inflammatory reactions [2]. These reactions, which are secondary immune responses, typically manifest 2–4 weeks post‐injection as local granulomatous reactions or non‐specific infections, clinically presenting as localized erythema, tissue induration, or painful nodules, and are often misdiagnosed [3]. Among these, granulomatous perioral dermatitis, a distinct variant of perioral dermatitis, with unknown etiology, is characterized by monomorphic papules localized to the perioral, periorbital, and perinasal areas. It is more prevalent in children and may be associated with the long‐term use of corticosteroids, potentially due to their ability to disrupt skin barrier function and exert immunosuppressive effects [4]. Here, we report a case of granulomatous perioral dermatitis‐like lesions following nasolabial furrow HA injection.

## 1 Case Presentation

**FIGURE 1:**
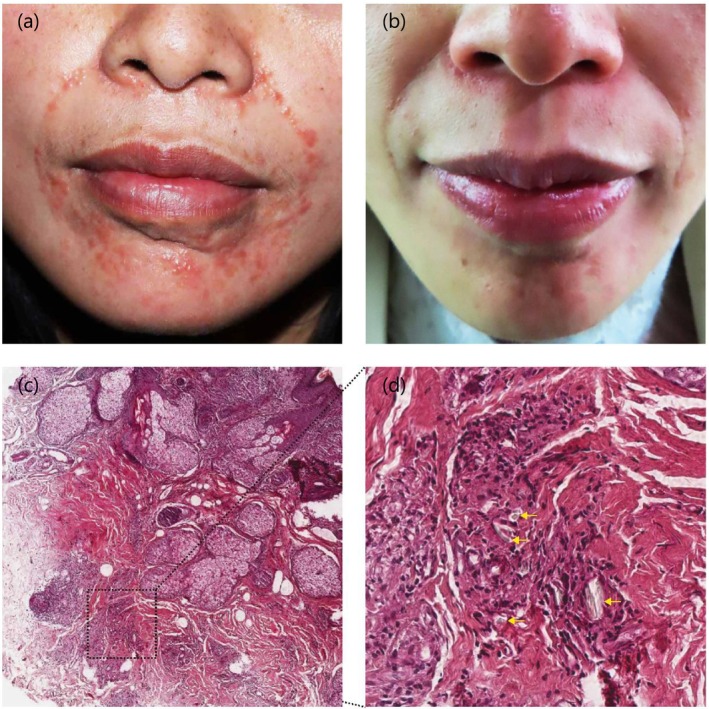
(a) Erythema and dense papules in bilateral nasolabial sulcus and mandible. (b) Skin lesions improved after isotretinoin treatment. (c) Chin biopsy showing granulomatous inflammatory infiltrate of histiocytes, lymphocytes, and giant cells in the dermis (hematoxylin and eosin, ×5 original magnification). (d) Arrows pointing to amorphous substance deposition (hematoxylin and eosin, ×20 original magnification).

A 39‐year‐old female patient presented to our department with erythema in the nasolabial folds and perioral area accompanied by persistent itching for 2 months. Three months prior, she had undergone bilateral nasolabial furrow wrinkle treatment at another medical institution, receiving a total of 2 mL HA (Natural, Bloomage Biotechnology). No fever or signs of infection occurred post‐injection. One month following the injection, she developed progressively worsening erythema and papules in the treated areas, with no exudate or erosion, along with persistent pruritus and prickling pain (Figure 1a). Initial treatment with topical mometasone furoate for 1 week at another hospital yielded no improvement. The patient was in good general health and had no relevant family medical history.

Systemic examination revealed no abnormalities. Dermatological examination revealed varying sizes of erythema in the bilateral nasolabial folds and perioral area, accompanied by ill‐defined dense papules with partial coalescence. The surrounding skin temperature was within normal limits.

Initial treatment of oral doxycycline capsule (4 mg/kg/d) for 4 weeks showed no improvement. A skin biopsy was performed, and oral isotretinoin at a dosage of 0.4 mg/kg/d (10 mg twice daily) was initiated following doxycycline discontinuation. After 4 weeks of isotretinoin therapy, the perioral lesion resolved completely without adverse effects, preserving the aesthetic results of HA injection (Figure 1b).

Histopathological examination of the chin lesion revealed mild epidermal hyperplasia and mixed inflammatory cells, predominantly infiltrated by histiocytes and lymphocytes within the dermal tissues, with sparse multinuclear giant cells and basophilic amorphous deposits (Figure 1c,d). The pathological diagnosis indicated foreign body granuloma. The histochemical stain for fungal organisms was negative.

## 2 Discussion

The immunological mechanism of foreign body granuloma remains unclear, potentially influenced by filler material properties, dose, and host immune status. Variability in individual responses and clinical manifestations often complicates treatment, leading to refractory or recurrent lesions [5]. Intriguingly, in this rare case, granulomatous inflammation occurred not only at the injection site but also extended to the submental and chin areas. The phenotype of these lesions, along with prior corticosteroid use, raises perioral dermatitis as a diagnostic consideration. However, the detection of filler material in non‐injection site pathology suggests that the foreign body granulomatous reaction may spread beyond the local region, differing from typical perioral dermatitis. The formation of distant foreign body granulomas may be associated with filler migration, inflammatory cell infiltration, and the unique physiologic architecture of perioral muscular tissues [6]. In this case of granuloma perioral dermatitis‐like lesions unresponsive to local mometasone furoate and oral doxycycline, low‐dose isotretinoin demonstrated favorable therapeutic efficacy. These findings suggest that for a localized or adjacent foreign body granulomatous reaction, rather than a systemic immune response to cosmetic fillers, isotretinoin therapy is more efficacious.

## Ethics Statement

The authors have nothing to report.

## Consent

The patient's consent was obtained for the publication of the case details and any accompanying images in this manuscript.

## Conflicts of Interest

The authors declare no conflicts of interest.

## Data Availability

Data sharing not applicable to this article as no datasets were generated or analysed during the current study.
